# Real world experience of patients with amyotrophic lateral sclerosis (ALS) in the treatment of spasticity using tetrahydrocannabinol:cannabidiol (THC:CBD)

**DOI:** 10.1186/s12883-019-1443-y

**Published:** 2019-09-07

**Authors:** Thomas Meyer, Andreas Funke, Christoph Münch, Dagmar Kettemann, André Maier, Bertram Walter, Annett Thomas, Susanne Spittel

**Affiliations:** 1 Centre for ALS and other motor neuron disorders, Charité – Universitätsmedizin Berlin, corporate member of Freie Universität Berlin, Humboldt-Universität zu Berlin, and Berlin Institute of Health, Berlin, Germany; 2 Ambulanzpartner Soziotechnologie APST GmbH, Westhafenstr. 1, 13353 Berlin, Germany; 3 Neurologische Facharztpraxis, Lessingstraße 24, 15745 Wildau, Germany

**Keywords:** Amyotrophic lateral sclerosis, Tetrahydrocannabinol, THC, Cannabidiol, CBD, Spasticity, Patient experience, Treatment satisfaction questionnaire for medication, Net promotor score

## Abstract

**Background:**

Treatment of spasticity poses a major challenge in amyotrophic lateral sclerosis (ALS) patient management. Delta-9-tetrahydrocannabinol (THC):cannabidiol (CBD) oromucosal spray (THC:CBD), approved for the treatment of spasticity in multiple sclerosis, serves as a complementary off-label treatment option in ALS-related spasticity. However, few structured data are available on THC:CBD in the treatment of spasticity in ALS.

**Method:**

A retrospective mono-centric cohort study was realised in 32 patients that meet the following criteria: 1) diagnosis of ALS, 2) ALS-related spasticity; 3) treatment with THC:CBD. Spasticity was rated using the Numeric Rating Scale (NRS). Patient’s experience with THC:CBD was assessed using the net promoter score (NPS) and treatment satisfaction questionnaire for medication (TSMQ-9) as captured through telephone survey or online assessment.

**Results:**

The mean dose THC:CBD were 5.5 daily actuations (range < 1 to 20). Three subgroups of patients were identified: 1) high-dose daily use (≥ 7 daily actuations, 34%, *n* = 11), 2) low-dose daily use (< 7 daily actuations, 50%, *n* = 16), 3) infrequent use (< 1 daily actuation, 16%, *n* = 5). Overall NPS was + 4.9 (values above 0 express a positive recommendation to fellow patients). Remarkably, patients with moderate to severe spasticity (NRS ≥ 4) reported a high recommendation rate (NPS: + 29) in contrast to patients with mild spasticity (NRS < 4; NPS: − 44). For the three main domains of TSQM-9 high mean satisfaction levels were found (maximum value 100): effectiveness 70.5 (±22.3), convenience 76.6 (±23.3) and global satisfaction 75.0 (±24.7).

**Conclusion:**

THC:CBD is used in a wide dose range suggesting that the drug was applied on the basis of individual patients’ needs and preferences. Contributing to this notion, moderate to severe spasticity was associated with an elevated number of daily THC:CBD actuations and stronger recommendation rate (NPS) as compared to patients with mild spasticity. Overall, treatment satisfaction (TSQM-9) was high. The results suggest that THC:CBD may serve as a valuable addition in the spectrum of symptomatic therapy in ALS. However, prospective studies and head-to-head comparisons to other spasticity medications are of interest to further explore the effectiveness of THC:CBD in the management of spasticity, and other ALS-related symptoms.

**Electronic supplementary material:**

The online version of this article (10.1186/s12883-019-1443-y) contains supplementary material, which is available to authorized users.

## Background

Amyotrophic lateral sclerosis (ALS) is a severe, progressive and incurable neurodegenerative disorder of the upper and lower motor neurons [1, 2]. Patients with predominant upper motor neuron degeneration present with spasticity that is found in 40% of all ALS patients [3]. Spasticity is defined as a velocity-dependent increase in muscle tone in response to an externally imposed stretch or during voluntary movement. Spasticity of the extremities, trunk and bulbar region is associated with central paresis of upper and lower limbs, impaired trunk function, and a pseudobulbar syndrome leading to dysarthria and dysphagia. Beyond the functional effects in mobility, the increase in the muscle tone of spastic muscle groups is perceived as debilitating. It can cause muscle fibrosis, joint contractures, muscle cramps, and pain [4].

The treatment of spasticity is a main challenge in ALS treatment. Baclofen, tizanidine, and benzodiazepines are used to reduce spasticity in patients with ALS. However, these drugs demonstrate considerable limitations in efficacy and tolerability. One of the particular difficulties in ALS is in the combination of spasticity with lower motor neuron symptoms, which can be aggravated by spasticity regimes. Due to these challenges and limitations, there is a great demand for other pharmacological treatment options for spasticity associated with ALS [5–8]. In the past decade, several clinical studies have shown the safety and efficacy of tetrahydrocannabinol (THC) and cannabidiol (CBD) in the control of spasticity in people with multiple sclerosis (MS). THC:CBD (brand name Sativex) was approved for the treatment of spastic symptoms in patients with MS in 2011. This drug is a mixture of botanical extracts from the flowers and leaves of the hemp plant *Cannabis sativa L., folium* cum *flore*, which contains standardised concentrations of THC and CBD [9–11]. Given the efficacy and availability of THC:CBD in MS, this drug is increasingly being used off-label for spasticity treatment of persons with ALS.

In ALS, THC:CBD is used mostly as escalation therapy if symptoms cannot be adequately controlled or palliation achieved with baclofen or tizanidine. Only recently, a first phase 2 study provided preliminary evidence of efficacy and safety of THC:CBD compared with placebo in controlling spasticity in ALS [12]. This trial showed significant improvements in scores on the Modified Ashworth Scale, and some evidence of an additional beneficial effect on spasticity-related pain. Despite the off-label use of THC:CBD in ALS since 2011, and the emerging evidence for efficacy in ALS-associated spasticity so far few structured data are available on patient reported outcomes of ALS patients who received spasticity treatment with THC:CBD. The aim of this registry study was to explore the real world experience in the use of a standardised oromucosal spray containing THC:CBD for symptomatic treatment of spasticity related to ALS.

## Methods

### Study design

The observational study was conducted as a retrospective, monocentric, cross-sectional cohort study. The investigation was reported according to the STROBE criteria [13].

### Participants

Subjects who participated in the cohort study met the following inclusion criteria: 1) diagnosis of ALS (ICD-10 G12.2) according to the revised El Escorial criteria [14]; 2) presence of spasticity; 3) treatment with THC:CBD oromucosal spray (Sativex®); 4) consent to participate in the study; 5) participation in a case management program for ALS medication; 6) consent in electronic data capture using a digital research platform [15].

### Setting

Data were collected among ALS patients who were treated in a tertiary ALS centre in Berlin (Germany). The investigation was confined to patients treated with THC:CBD between May 2016 and September 2017. ALS trained neurologists confirmed the diagnosis of ALS according to the El Escorial criteria and made the indication for the treatment with THC:CBD. The anatomic region and severity of spasticity were classified by the neurologist (observer reported outcome). The physician’s classification of spasticity was assessed by the same investigator. The patient’s perception of spasticity (patient reported outcome) was obtained during the course of treatment. Before the patient’s assessment an instruction to the method of numeric rating scale (NRS) was performed by the investigator. Medication data on THC:CBD and other antispasmodic drugs were recorded on the basis of prescription data tracked on the APST platform. Data entry of prescription data was performed by data managers trained in the digital capture of medication data. THC:CBD was delivered via a highly standardised pump action oromucosal spray. Each 100 μL actuation contained 2.7 mg THC and 2.5 mg CBD in a 50:50 solution of ethanol and propylene glycol.

#### Data capture

All observed data including demographic data, clinical characteristics, medication data as well as scales and scores were captured on a digital portal named "APST platform (www.ambulanzpartner.de) [15]. Since all patients were registered on the digital platform, online self-assessment of scales and scores was encouraged. However, due to neurological and psychosocial limitations, online assessment was not possible for all participants. In these cases, the self-assessment data were primarily collected in a telephone interview by the investigator and subsequently captured on the platform.

### Variables

#### Demographic data and clinical characteristics

The following demographic data and clinical characteristics were collected for all participants: age, sex, diagnosis, type of onset (bulbar vs spinal onset), time since onset of symptoms, ALS severity as measured by the ALS functional rating scale revised (ALS-FRSr), cognitive impairment, region and severity of spasticity, and antispasmodic medication. An overview of demographic data and clinical characteristics is provided in Table 1 and the Additional file 1.

**Table 1:** Demographical and clinical characteristics of the study participants

Characteristics	Classification	Value
Gender	female, male, n (%)	25 (56.8), 19 (43.2)
Age in years	mean (SD)	57.3 (± 15.3)
Type of onset	spinal, bulbar, n (%)	42 (95.5), 2 (4.5)
Disease duration (months)	mean (SD)	58.4 (± 45)
ALS Functional Rating Scale revised	mean (SD)	23.0 (± 9.2)
Presence of cognitive impairment	n (%)	3 (6.8)

*n* Number of patients; *SD* standard deviation

#### Numeric rating scale (NRS)

The patient’s perception of spasticity and of spasticity-associated pain and cramps was recorded on the NRS, a one-dimensional assessment tool for recording the intensity of a symptom [16–19]. The 11-point scale ranges from 0 (no complaints) to 10 (worst imaginable complaints). The complaints are classified into four groups:

*None: 0 points.*

*Mild: 1 to 3 points.*

*Moderate: 4 to 6 points.*

*Severe: 7 to 10 points.*

#### Treatment with THC:CBD and other antispasmodic medication

##### Dose of THC:CBD

For THC:CBD, the number of actuations per day, each containing 2.7 mg THC and 2.5 mg CBD, was documented.

##### Discontinuation of THC:CBD

The date of discontinuation of THC:CBD treatment was recorded. The clinical characteristics of individual patients who terminated THC:CBD is provided in the Additional file 1. Adverse effects of THC:CBD and causes for the discontinuation of drug treatment were not collected within the scope of this study.

##### Antispasmodic medication other than THC:CBD

Data on the prescription (generic name, prescribed dose) of antispasmodic drugs other than THC:CBD were recorded. The treatment with baclofen, tizanidin, dantamacrin and botulinum toxin (in combination with THC:CBD) is provided in the Additional file 1.

#### Net promoter score (NPS)

The NPS was used for examining the patients’ attitude towards their treatment with THC:CBD. This metric is calculated based on responses to a single question: “How likely is it that you would recommend THC:CBD to a friend or colleague who suffers from ALS and spasticity?” The answers were rated on a numeric scale between 0 (absolutely unlikely recommendation) and 10 (highest likelihood of recommendation) points. The scores were assessed based on the following classification [20, 21]:

*10 or 9 points: Likely recommendation:*

*8 or 7 points: Indifferent recommendation:*

*6 to 0 points: Unlikely recommendation:*

Patients who respond with a score of 9 to 10 are considered as “promoters”. Those who rate the medication with 7 or 8 are classified as “indifferent”. The group of patients who express an unlikely recommendation (6 to 0 points) are defined as “detractors”. The NPS is calculated by subtracting the percentage of patients who are detractors from the percentage of patients who are promoters. Indifferent patients count toward the total number of respondents, thus decreasing the percentage of detractors and promoters. The NPS is the difference between persons with likely and unlikely recommendation, which is calculated as follows:

*NPS = “promoters” (in % of all patients surveyed) minus “detractors” (in % of all patients surveyed).*

The range of values of the NPS is between positive (+) 100 and negative (−) 100. An NPS with a positive score (greater than zero) is regarded as a supporting recommendation. An NPS of + 50 is considered as excellent [19, 20].

#### Treatment satisfaction questionnaire for medication (TSQM-9)

Satisfaction with THC:CBD treatment was assessed by means of TSQM-9. This score is a validated assessment scale containing nine questions concerning patients’ satisfaction with medication [22, 23]. TSQM-9 was also validated for the German language [24]. The questions are answered on a five-point or seven-point scale (for example, from very dissatisfied to very satisfied). Each of the nine questions is evaluated in a total score that can range from 0 to 100. A higher total score equates to greater satisfaction. The total score is calculated as follows:

*Total score for question X = ((response score of question X minus 1) ÷ (highest possible response score minus the lowest possible response score) multiplied by 100).*

The questions of TSQM-9 refer to three dimensions: effectiveness (questions 1 to 3), convenience (questions 4 to 6) and global satisfaction (questions 7 to 9). In addition to the calculation of the total score for the nine individual questions of TSQM-9, a score can also be calculated for the three dimensions of effectiveness, convenience and global satisfaction. In this calculation also, the total score of the three dimensions can be between 0 and 100. A higher total score equates to greater satisfaction. The total scores for effectiveness, convenience and global satisfaction are calculated as follows:

*Total score for effectiveness: ([sum (response score for question 1 plus question 2 plus question 3) minus 3] divided by 18) multiplied by 100.*

*Total score for convenience: ([sum (response score for question 4 plus question 5 plus question 6) minus 3] divided by 18) multiplied by 100.*

*Total score for global satisfaction: ([sum (response score for question 7 plus question 8 plus question 9) minus 3] divided by 14) multiplied by 100.*

### Protocol approvals and registrations

The study protocol was approved by the Medical Ethics Committee of the Charité – Universitätsmedizin Berlin, Germany under the number EA1/219/15. A signed patient information and informed consent form was obtained from all participating patients.

### Data analysis

Descriptive statistics were used for the statistical analysis (frequency in percent, mean, median, standard deviation in ±). Differences of frequencies between subgroups were assessed by Fisher’s exact test or Chi-square test. *P* values have been reported with a 95% confidence interval. The data were analysed using SPSS (version 24.0).

## Results

### Sample characteristics

**Fig. 1:**
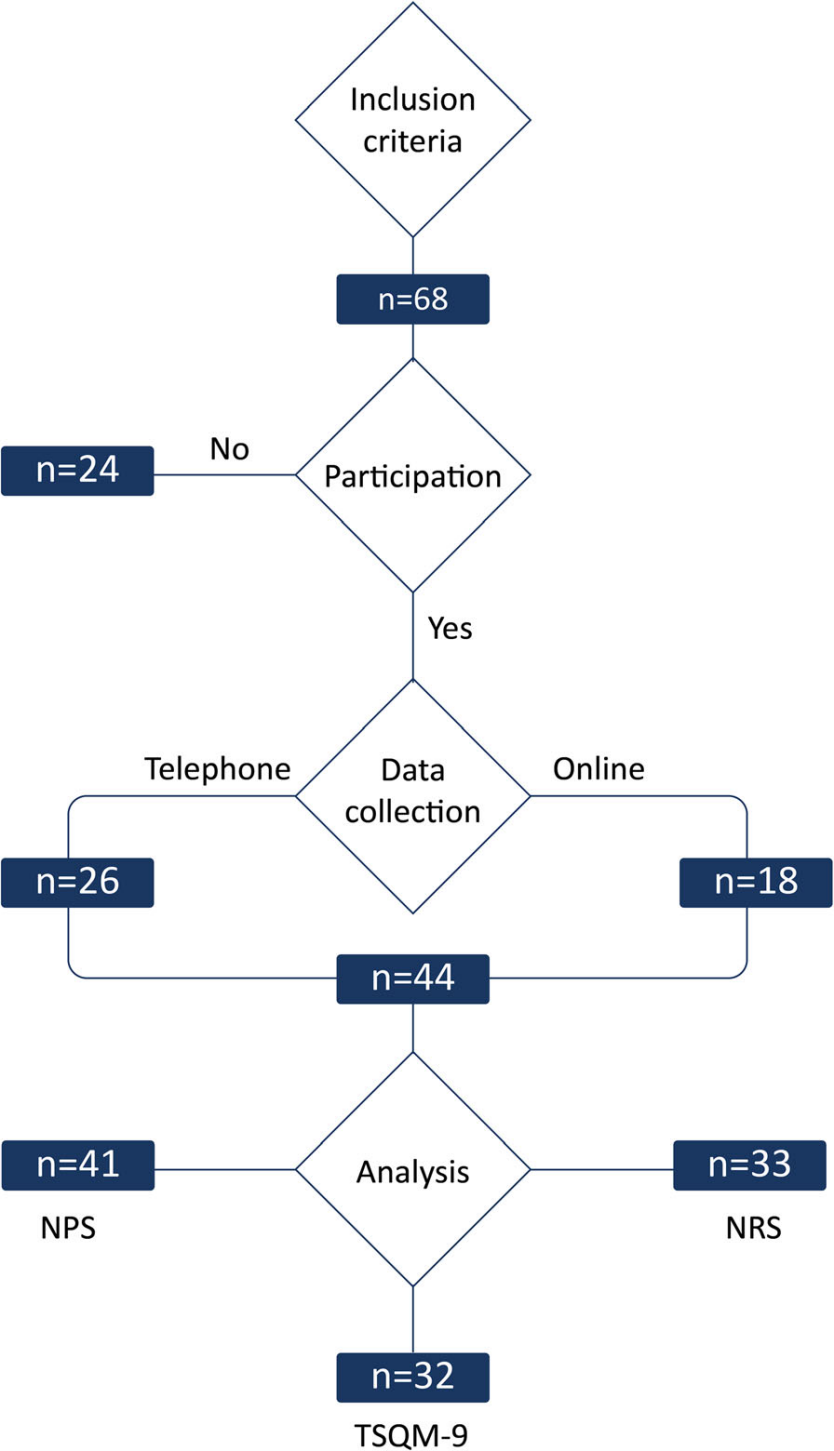
Sample characteristics. A total number of ALS patients at the study site fullfilling the inclusion criteria were invited to participate in the survey. A subgroup responded and participated in the study. Data were collected online or via telephone survey. Data sets for severity of spasticity (Numeric Rating Scale, NRS), recommendation of treatment (Net Promoter Score, NPS) and treatment satisfaction (Treatment Satisfaction Questionnaire for Medication, TSQM-9) were obtained. n = number of patients and analysable datasets, respectively

A total of 68 patients with ALS were invited to participate in the questionnaire. 44 patients participated in the study (65%). Data sets were collected through online assessment (*n* = 18) or in a telephone survey (*n* = 26). One patient, being completely paralysed (ALS-FRSr = 0 of 48 score points), used an eye-controlled communication device for completion of the questionnaire. In the cohort of 44 patients, a complete dataset was obtained for 32 subjects (Fig. 1).

### Demographic data and clinical characteristics

Of the patients surveyed, 56.8% (*n* = 25) were women and 43.2% (*n* = 19) were men. At the time of the survey, the average age of the respondents was 57.3 years (± 15.3; median: 57.5). The majority of participants (56.8%; *n =* 25) were between 41 and 70 years old. Twenty-five percent (*n* = 11) were older than 70 years. At the time of the survey, the youngest patient was 27 years old and the oldest patient was 87 years. Mean disease duration since symptom onset was 58.4 months (± 45 months; median: 42 months). 95.5% of the patients showed a spinal symptom onset. The ALS-FRS score was 23 scale points (± 9.2 scale points) as compared to the maximum number of 48 scale points. In 6.8% of the patients (*n* = 3) clinical signs of frontotemporal lobe degeneration were described. A summary of demographic and clinical data is summarised in Table 1. The clinical characteristics of individual patients is provided in the Additional file 1.

### Location and severity of spasticity

**Fig. 2:**
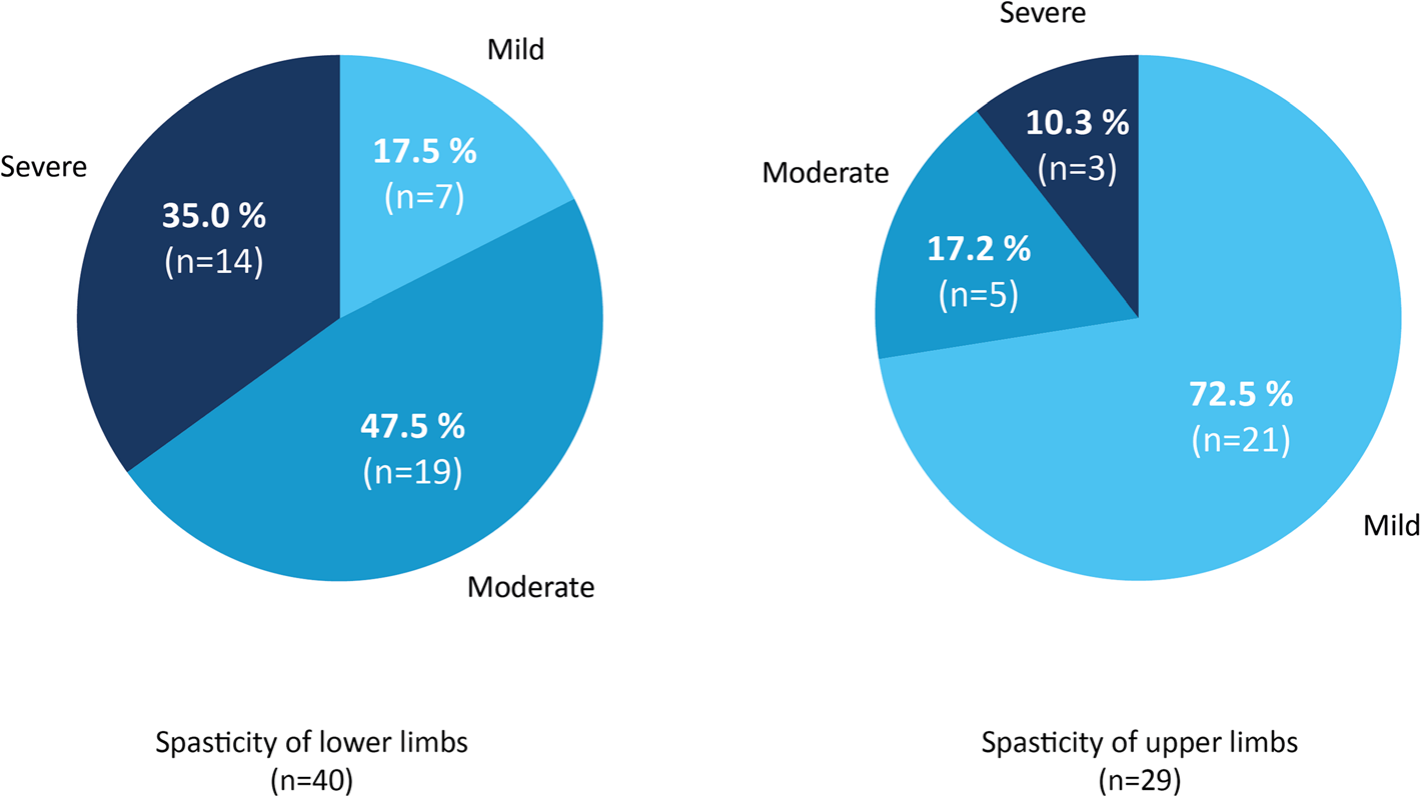
Location and severity of spasticity**.** The location of spasticity in terms of upper and lower limb involvement is shown. The severity of spasticity, as assessed by neurologists, was classified into severe, moderate and mild spasticity. It was recorded on a 4-point scale and classified as follows: severe spasticity: 4 points, moderate spasticity: 3 points, mild spasticity: 2 points, no spasticity: 1 point

The majority of the patients in the study (95.5%; *n* = 42) showed spasticity of the lower extremities, either limited to the legs or in combination to spasticity of the upper extremities. 29.5% of the patients (*n* = 13) had only spasticity in the lower extremities, all of whom had a spinal onset. Spasticity of the arms, either limited to the arms or in combination to spasticity of the lower limbs, was found in 68.2% (*n* = 30) of patients. Only one of the study patients showed spasticity confined to the upper extremities (2.3%). In a classification of severity of spasticity into severe, moderate and mild, there were regional differences in the severity level of spasticity. In the lower extremities, severe spasticity was frequently described (33.3% of patients). A contrasting constellation was found in the arms: Severe spasticity was rarely demonstrated (7.1%). In the arms mild spasticity prevailed (72.5%), in contrast to the lower limbs were mild severity was rarely found (17.5%; Fig. 2).

### Perception of spasticity

Most of the patients considered spasticity as a severe (24.2%, *n* = 8) or moderate (48.5%, *n* = 16). A small number of patients (6.1%; *n* = 2) had no perception of spasticity. In those patients, spasticity was demonstrated in the neurologist’s investigation, whereas the patient had no subjective perception for the symptom.

### Perception of pain and cramps

Spasticity-associated pain and cramps were a common symptom in the examined cohort. Of the surveyed ALS patients, 70% (*n* = 23) reported pain. In 51.5% (*n* = 17), the pain was moderate to severe. 84.4% of patients (*n* = 27) reported cramps. In 59.4% (*n* = 19), the cramps were moderate to severe.

### Treatment with THC:CBD and other antispasmodic medication

#### Dose range of THC:CBD

**Fig. 3:**
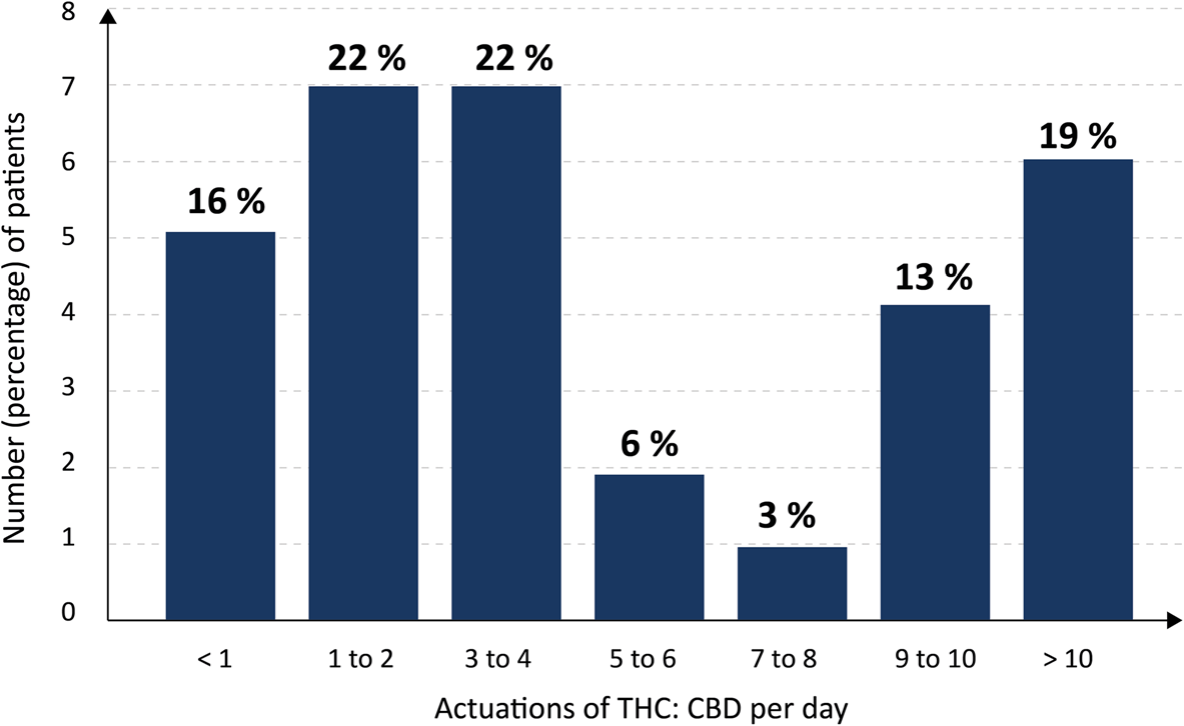
Dose distribution in the use of THC:CBD. Maximum number of actuations per day. The daily dose of THC:CBD, as assessed by the maximum number of actuations per day, is shown. Number of patients = 32

The mean dose THC-CBD were 5.5 daily actuations (± 5.1; range < 1 to 20). The daily dose of THC:CBD, as assessed by the number of actuations per day, showed a wide range among the studied patients (Fig. 3). Three distinct subgroups of patients were identified: 1) high-dose daily use (7 or more daily actuations, 34% of patients, *n* = 11), 2) low-dose daily use (six or less daily actuations, 50% of patients, *n* = 16), 3) low-dose less then daily use (less than one daily actuation, 16% of patients, *n* = 5). 6.2% of the studied patients (*n* = 2) used more than 12 and up to 20 actuations per day.

#### Discontinuation of THC:CBD

40% of the studied patients (*n* = 16) discontinued THC:CBD treatment during the observation period.

#### Dose of THC:CBD in relation to severity of spasticity

**Fig. 4:**
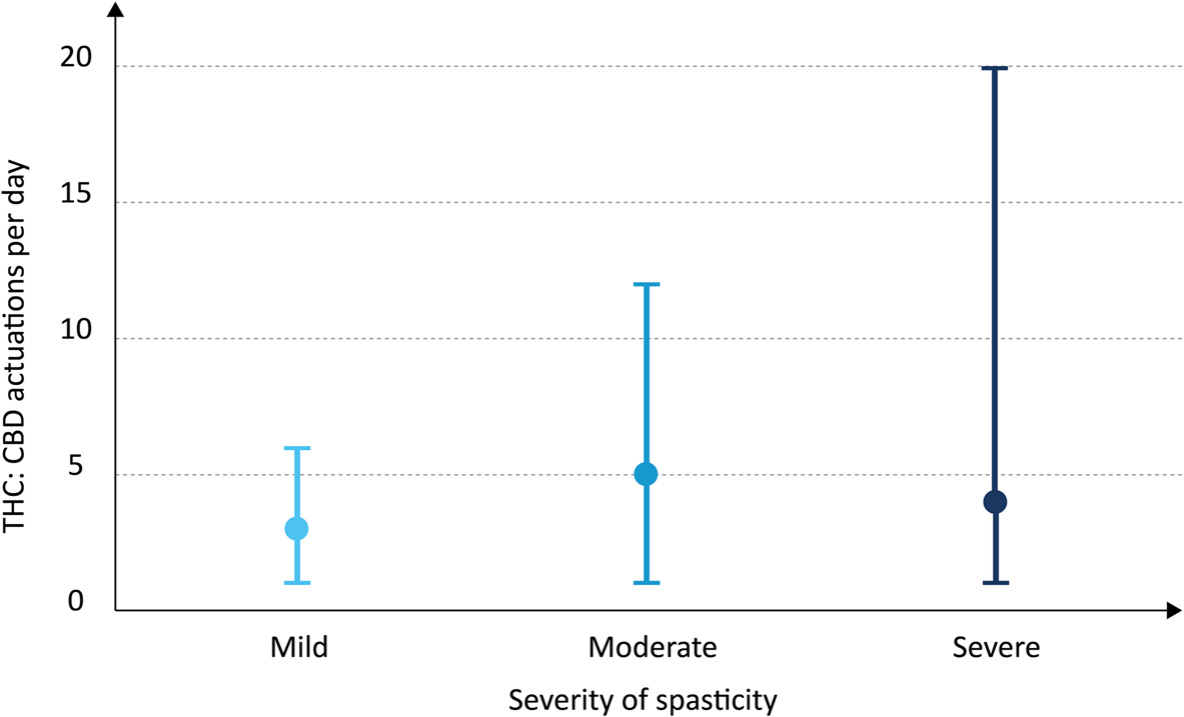
Dose of THC:CBD in relation to severity of spasticity. THC:CBD dose was defined as maximum number of THC:CBD actuations per day (mean, maximum and minimum number). Severity of spasticity was assessed by neurologists using a 4-point scale: no spasticity (1 point); mild spasticity (2 points); moderate spasticity (3 points); severe spasticity (4 points). Data are shown for spasticity of the lower extremities. Number of patients = 32

For the treatment of lower limb spasticity, a correlation of symptom severity and the applied THC:CBD dose was found (Fig. 4). In patients with severe spasticity, a mean number of 7.3 actuations (± 6.0) were used, whereas in patients with mild spasticity 3.3 daily actuations (± 5.8) were reported.

#### Antispasmodic medication other than THC:CBD

25% of patients (*n* = 10) received other antispasmodic medication in combination with THC:CBD. Baclofen was used in 25% of patients (*n* = 10) whereas tizanidine was observed in 5% of studied individuals (*n* = 2). None of the patients received dantamacrin or botulinum toxin in combination with THC:CBD.

### Net promoter score (NPS)

**Fig. 5:**
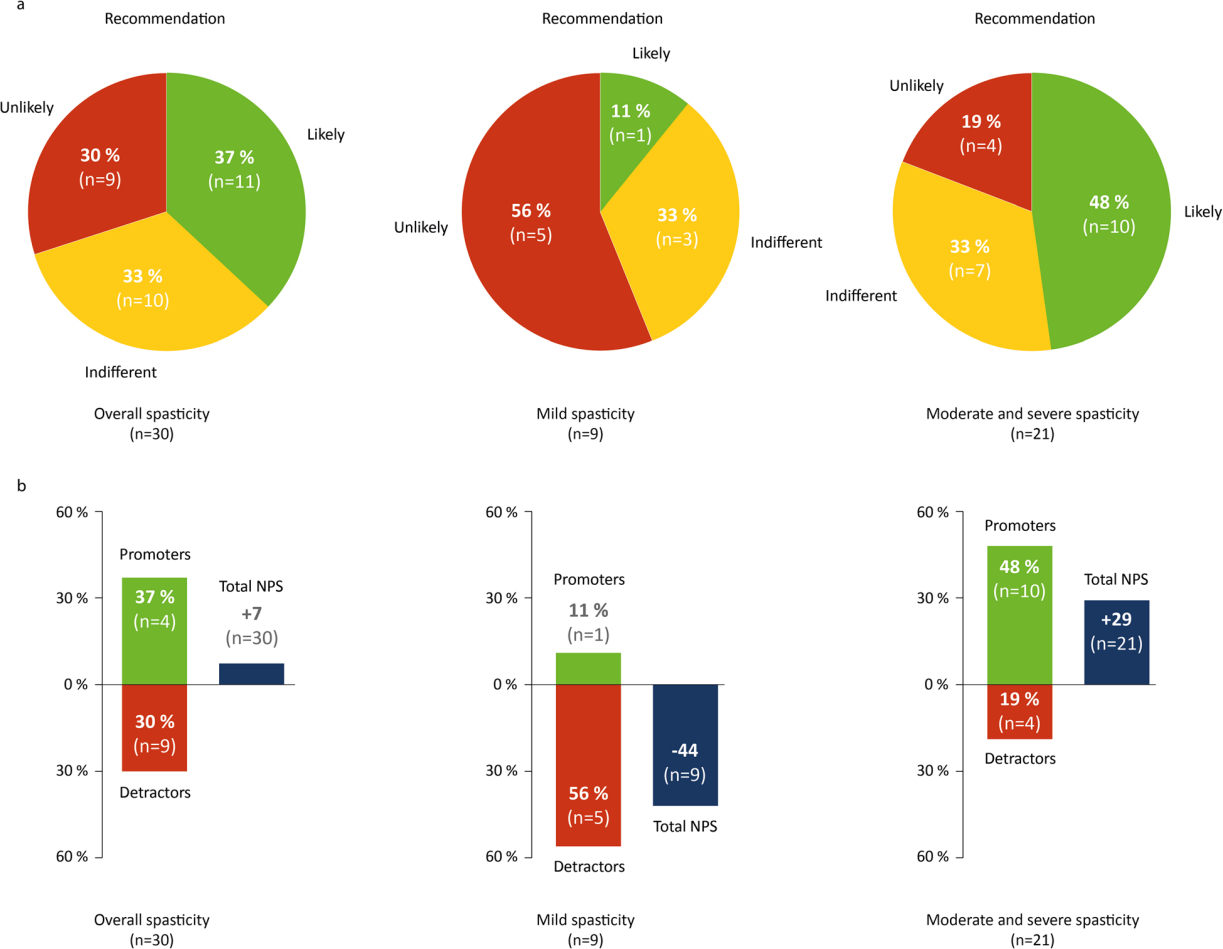
Patients’ recommendation of THC:CBD overall and in relation to severity of spasticity. The likelihood of recommendation of THC:CBD in the overall patient group and in relation to the severity of spasticity (**a**) was assessed using the Net Promoter Score (NPS): unlikely recommendation (0 to 6 points); indifferent recommendation (7 to 8 points); likely recommendation (9 to 10 points). The patients’ perception of spasticity was assessed using the Numeric Rating Scale (NRS): Patients with moderate to severe spasticity (NRS ≥ 4), patients with mild or no spasticity (NRS < 4). The total NPS (**b**) is calculated by subtracting the percentage of patients who are detractors from the percentage of patients who are promoters. Patients who respond with a score of 9 to 10 are considered as “promoters”. Those who rate the medication with 7 or 8 are classified as “indifferent” (not shown in **b**). The group of patients who express an unlikely recommendation (6 to 0 points) are defined as “detractors”. *n* = number of patients

The patient’s attitude towards their treatment with THC:CBD, as assessed by NPS, is shown in Fig. 5. The overall NPS score was + 4.9 points. This evaluation is considered a positive attitude of patients towards THC:CBD as a NPS higher than zero is generally deemed good [20]. Remarkably, patients with moderate to severe spasticity (NRS ≥ 4) had a very high likelihood (NPS: + 28) to recommend THC:CBD, just in contrast to patients with mild spasticity (NRS < 4; NPS: − 44).

### Treatment satisfaction questionnaire for medication (TSQM-9)

#### Detailed results for individual TSQM-9 questions

**Fig. 6:**
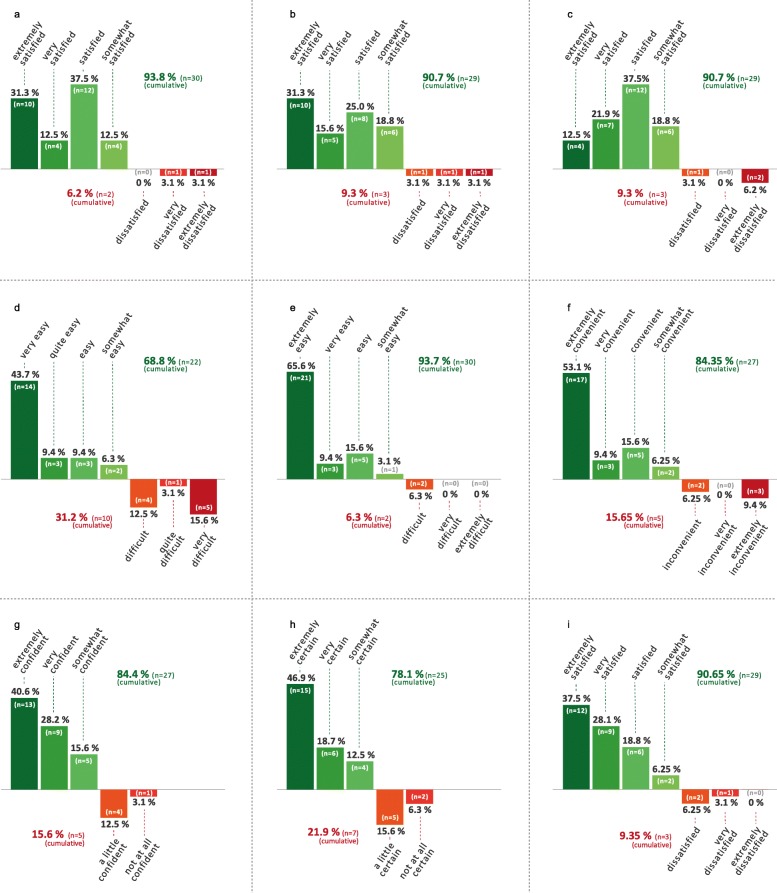
The patient’s treatment satisfaction with THC:CBD, as assessed by Treatment Satisfaction Questionnaire for Medication (TSQM-9). The score was evaluated separately in nine questions, which are as follows. Question 1 (**a**) – ability of THC:CBD to prevent or treat spasticity: “How satisfied or dissatisfied are you with the ability of THC:CBD to prevent or treat your spasticity?”; Question 2 (**b**) – the way THC:CBD relieves spasticity: “How satisfied or dissatisfied are you with the way THC:CBD relieves your symptoms?”; Question 3 (**c**) – amount of time it takes THC:CBD to start working: “How satisfied or dissatisfied are you with the amount of time it takes the medication to start working?”; Question 4 (**d**) – usability of THC:CBD: “How easy or difficult is it to use the medication in its current form?”; Question 5 (**e**) – planning when to use THC:CBD: “How easy or difficult is it to plan when you will use the medication each time?”; Question 6 (**f**) – administration of THC:CBD as instructed: “How convenient or inconvenient is it to take the medication as instructed?”; Question 7 (**g**) – taking THC:CBD is a good thing: “Overall, how confident are you that taking this medication is a good thing for you?”; Question 8 (**h**) – the good things about THC:CBD outweigh the bad things: “How satisfied are you that the good things about this medication outweigh the bad things?”; Question 9 (**i**) – overall satisfaction THC:CBD: “Taking all things into account, how satisfied or dissatisfied are you with this medication”; *n* = number of patients

The patients’ treatment satisfaction with THC:CBD, as assessed by TSQM-9, is shown in Fig. 6. The score was evaluated separately in the nine addressed questions, which are as follows.

TSQM-9, Question 1 (effectiveness) – ability of THC:CBD to prevent or treat spasticity: 94% of patients (*n* = 30) were reasonably to very satisfied with the ability of THC:CBD for the treatment of spasticity.

TSQM-9, Question 2 (effectiveness) – the way THC:CBD relieves spasticity: 91% of patients (*n* = 29) were reasonably satisfied to very satisfied with the way how THC:CBD alleviated the symptoms of spasticity.

TSQM-9, Question 3 (effectiveness) – amount of time it takes THC:CBD to start working: 91% of patients (*n =* 29) were reasonably satisfied to very satisfied with the time it takes THC:CBD to alleviate spasticity.

TSQM-9, Question 4 (convenience) –usability of THC:CBD: 69% of patients (*n* = 22) considered the use of THC:CBD in its current form as being relatively easy to very easy. To be noted, however, there was a substantial percentage in the cohort (31%; *n* = 10) regarding the use of THC:CBD as difficult.

TSQM-9, Question 5 (convenience) –planning when to use THC:CBD: 94% of patients (*n =* 30) considered the plan when to use THC:CBD each time as being relatively easy to very easy.

TSQM-9, Question 6 (convenience) – administration of THC:CBD as instructed: 84% of patients (*n* = 27) considered the administration of THC:CBD as instructed as being relatively easy to very easy. However, there was a substantial percentage in the cohort (16%; *n* = 5) regarding the administration of THC:CBD as inconvenient or very inconvenient.

TSQM-9, Question 7 (global satisfaction) – taking THC:CBD is a good thing: 84% of patients (*n =* 27) were relatively convinced to very convinced that taking THC:CBD was a good thing for the patient. Remarkably, 16% of patients (*n* = 5) took THC:CBD even though they were not completely convinced or not all convinced of the medication.

TSQM-9, Question 8 (global satisfaction) – the good things about THC:CBD outweigh the bad things: 78% of patients (*n* = 25) were relatively certain to very certain that the good things about THC:CBD outweigh the bad things. To be noted, however, there was a substantial percentage in the cohort (22%; *n* = 7) take used THC:CBD even though they were not completely certain or not at all certain that the positive aspects outweigh the negative side of the medication.

TSQM-9, Question 9 (global satisfaction) – overall satisfaction THC:CBD: 91% of patients (*n* = 29) were, taking all things into account, reasonably satisfied to very satisfied with the medication of THC:CBD.

#### Total score for individual TSQM-9 questions

The total score for all TSQM-9 items (questions 1 to 9) is shown in Table 2. The highest satisfaction was found for the planning when to use THC:CBD (question 5; total score 87.5; ± 20.3) and overall satisfaction (question 9; total score: 79.2; ± 22.8). Reduced satisfaction was identified in the usability of THC:CBD (question 4; total score: 65.6; ± 38.6) and in the latency of THC:CBD to start working (question 3; total score: 66.2; ± 24.1).

**Table 2:** Total score of Treatment Satisfaction Questionnaire for Medication (TSQM-9)

Questions	Q 1	Q 2	Q3	Q4	Q5	Q6	Q7	Q8	Q9
Mean Score	73.5	71.9	66.2	65.6	87.5	76.6	72.7	71.1	79.2
STD	24.6	26.2	24.1	38.6	20.3	32.5	29. 3	33.7	22.8

The patients’ treatment satisfaction with THC:CBD, as assessed by Treatment Satisfaction Questionnaire for Medication (TSQM-9). The score was evaluated separately in nine questions. Each of the nine questions is evaluated in a total score that can range from 0 to 100. A higher total score equates to greater satisfaction. Question (Q) 1 – ability of THC:CBD to prevent or treat spasticity; Q 2 – the way THC:CBD relieves spasticity; Q 3 – amount of time it takes THC:CBD to start working; Q4 – usability of THC:CBD; Q 5 – planning when to use THC:CBD; Q 6 – administration of THC:CBD as instructed; Q 7 – taking THC:CBD is a good thing; Q 8 – the good things about THC:CBD outweigh the bad things; Q 9 – overall satisfaction THC:CBD; *n* number of patients. *STD* standard deviation

#### Total score for main TSQM-9 domains

For the three main domains of TSQM-9 high satisfaction levels were found, which are as follows: effectiveness – mean score 70.5, ± 22.3; convenience – mean score 76.6, ± 23.3; global satisfaction – mean score: 75.0, ± 24.7.

## Discussion

### Sample selection

The present report examines the treatment of ALS-related spasticity using THC:CBD. Systematic assessment of “real world experience” was facilitated by the use of a digital management platform [25]. Currently, more than 1.600 ALS patients are registered on that platform representing a substantial fraction of the ALS population in Germany [14]. The digitization of drug management allowed the tracking and systematic analysis of real world use of THC:CBD in ALS. Despite the advantages of the platform-based registry and substantial number of analysed patients with off-label use of THC:CBD several limitations have to be addressed. The study cohort was monocentric and covered a rather small sample size. Given the limited sample size the statistical analysis was therefore confined to descriptive methods. Furthermore, a subgroup of patients did not respond to the study invitation (*n* = 24; 35%). The reasons for non-responding was not explored systematically. Therefore, an observation bias in the cohort of participating patients – as compared to the group of patients who declined study participation – has to be considered. Furthermore, the platform-approach and the study site being a tertiary ALS centre may have created some further observation bias. Therefore, it is conceivable that more intensive THC:CBD treatment may be overrepresented in this cohort while drug treatment of less complex ALS phenotypes was provided independently from the platform, i. e. outside the analysed data set. Moreover, 7% of studied patients (*n* = 3) showed mild signs of frontotemporal lobe degeneration by means of a dysexecutive syndrome. Given the rather low grade of neuropsychological syndrome we opted to include the data of those patients. Therefore, some distortion of patient reported data cannot be excluded in this particular group of patients.

### Severity and perception of spasticity

Severity of spasticity was classified by the ALS-trained neurologists in three main categories (severe, moderate, mild). The physician’s assessment of spasticity was narrowed to these main graduations as functional aspects of treatment was not the emphasis of this study. However, in further investigations a more precise classification and functional assessments of spasticity (before and after initiation of THC:CBD treatment) are of main interest. The Ashworth Scale, which has been used in some previous ALS studies, was not applied given its limitations [6]. The score does not reflect the possible co-existence of spasticity with lower motor neuron involvement which is specific for ALS. Furthermore, this score might not represent the patients’ subjective perception of spasticity. In an attempt to overcome these limitations, a disease­specific self­reported scale, the ALS spasticity index (SI-ALS) has been developed. However, the SI-ALS was released only recently, well after our study had been completed [26]. In our study, both the neurologist’s classification of spasticity, and the patient’s subjective perception of spasticity were obtained. By that means, we found that most of the patients treated with THC:CBD perceived severe or moderate spasticity (60 to 77%, Fig. 3). In a smaller group of patients (14 to 23%, Fig. 3), low-grade spasticity was reported. It is uncertain whether those patients were treated with a-priori lower-grade spasticity or THC:CBD may have achieved symptom control in those patients. In principle, an therapeutic effect of THC:CBD is conceivable as in a recent phase 2 trial (CANALS study) THC:CBD was reported to reduce spasticity in ALS [12]. However, given the non-interventional study design, our data provided little opportunity to interpret the reduced muscle tonus as response to THC:CBD. Beyond spasticity, the perception of pain and muscle cramps was documented. Both spasticity-associated symptoms were highly prevalent in the studied cohort (70% pain; 84% muscle cramps). In other ALS cohorts, the prevalence of pain has been reported to be as high as 51–80% [27, 28]. Cannabinoids are increasingly recognised as a treatment option in neuropathic and non­neuropathic pain [29, 30]. However, a study of NPS and TSQM-9 in the context of ALS-related pain was not subject of this registry study.

### Dose of THC:CBD

Our study, to our knowledge, provided the first real world data (outside a clinical trial) on the dosing of THC:CBD in ALS. In the CANALS study, a mean dose of daily 8.2 actuations (SD 2.9; range 1 to 12) was reported [12]. In our cohort the mean dose was slightly lower (6 daily actuations). More importantly, a wider range in the daily dose was identified (± 4; range < 1 to 20). Basically, in the treatment with oromucosal spray of THC:CBD, patients were instructed to self-titrate during the first 14 treatment days following a predefined escalation regime to their optimal dose, up to 12 daily actuations, with the aim of balancing symptom control and unwanted effects. Despite this general instruction, a diversity of individual dose regimes was reported. For reasons of clarity, three distinct use pattern of THC:CBD were defined: 1) high-dose daily use (7 or more actuations, 34%), 2) low-dose daily use (6 to 1 daily actuations, 50%), 3) low-dose less than daily use (< 1 daily actuation, 16%). The distinction between high vs. low-dose use was made at the mean number 6 actuations per day. Patients of the high-dose group (34%) were treated with THC:CBD doses for that clinical trials in ALS and MS have demonstrated alleviation of spasticity [9–11]. Presumably, this patient group benefited most from THC:CBD treatment. A correlation of symptom severity and the applied THC:CBD dose was found (Fig. 4). In patients with severe lower limb spasticity, a mean number of 7.3 actuations (± 6.0) were used, whereas in patients with mild spasticity 3.5 (± 2.2) daily actuations were reported. In patients with low-dose daily use the efficiency of THC:CBD treatment is not excluded but yet uncertain as long there are no dose-range studies completed. In patients with less than daily use, an episodic use on demand in terms of escalation therapy of fluctuating spasticity is conceivable. However, the treatment modalities in this group of patients have not been studied yet. Further studies are needed to define the dose correlation and minimum effective dose of THC:CBD in ALS-related spasticity. Remarkably, 40% of the patients discontinued the treatment of THC:CBD within the observation period. So far, we have not identified demographic or clinical characteristics (such as age, disease duration or disease severity) that correlated with termination of THC:CBD (data shown in additional file 1). Reasons for discontinuation of treatment were not in the scope of this non-interventional cohort study. In future studies, a systematic analysis of adverse events and side effects is warranted in order to determine the benefit-risk profile of THC:CBD in the treatment of ALS-related spasticity.

### Recommendation of THC:CBD by patients

The NPS serves as a robust instrument for the assessment of products and services, which is used mostly outside of medicine [20, 21]. This likely-to-recommend question is considered to exhibit behaviour in response to user experience. Although the validation of this score in medicine is still limited, the NPS finds growing use in outcome research, mainly due to the simplicity of the method and the established calculation matrix [31, 32]. In the overall group of patients there was a NPS score of + 4.9 that translates into a moderately positive recommendation rate. Remarkably, between the patient groups of low-grad spasticity (NRS < 4) and of moderate to severe spasticity (NRS ≥ 4), there were noticeable differences in the NPS (*p* < .05). Patients with low-grade spasticity were not like to recommend THC:CBD to fellow patients (showing a negative NPS of − 44; Fig. 5). In contrast, patients with moderate to severe spasticity were highly likely to recommend the treatment (demonstrating a very positive NPS of + 28). Given the low case numbers, the significance in the differences of NPS between the two patient groups have to be interpreted with some caution. NPS data on THC:CBD or other medications have not yet been published in ALS. In particular, there are no comparative data for baclofen, tizanidine or other antispasmodic medication. The obtained NPS scores for THC:CBD are, therefore, to be regarded as a baseline for further studies.

### Treatment satisfaction

The evaluation of TSQM-9 showed a high overall level of satisfaction for THC:CBD treatment in most studied ALS patients (84% of patients gave positive results in question 9 for global satisfaction). However, like with NPS, the limitation with this score lied in the lack of comparative TSQM-9 data for other spasticity medications such as baclofen and tizanidine. This limitation addresses a fundamental problem in clinical ALS research where no systematic studies on patient reported outcomes of symptomatic and palliative medications have been published so far. Given the lack of comparative data, the outcomes from TSQM-9 are to be regarded as pilot data. The large proportion of patients stating high overall satisfaction with THC:CBD (91%, *n* = 29) somehow contrasts with smaller proportion of patients that is ready to recommend the treatment to fellow patients (37%, *n* = 11, positive NPS results). Apparently, TSQM-9 (satisfaction) and NPS (recommendation), seem to assess distinct aspects of patients’ experience. Although there is no comparative between TSQM-9 and NPS, it is well conceivable that the patient’s likeliness to recommend a therapy to other patients (NPS) is handled more critically and more stringently than the patient’s own satisfaction (TSQM-9). In a more analysis of TSQM-9, the score showed difficulties among ALS patients (31%; *n* = 10) in taking the medication in its current form (TSQM-9, question 4). This finding may reflect barriers in handling the oromucosal spray due to upper extremity paresis or due to difficulties in fully opening the mouth. The critical use of the medication THC:CBD underscores the necessity of instructions of patients and their relatives in order to facilitate the administration of the prescribed medication.

### Unsolved issues

A comparative analysis of THC:CBD with other antispasmodic medications, particularly baclofen and tizanidine, was not in the scope of this non-interventional study. However, controlled studies are needed to achieve a head-to-head comparison of THC:CBD to other antispasmodic medicines preferably in a multicentric design. Furthermore, a dose correlation of THC:CBD to symptom control is of interest. Given the palliative treatment aim in most ALS patients, symptom control may not necessarily relate to improvement in motor function. Beyond function, the various dimensions of spasticity such as muscle tone, pain, cramps, and mobility restrictions may be modified by THC:CBD [27, 28, 33]. Contributing to this notion, future studies, as our observational study, should touched upon treatment aims beyond functional endpoints. This view is supported by the results of a systematic analysis on physical therapy in ALS demonstrating an increasing patients’ treatment satisfaction despite the continuous decline of motor function [34].

## Conclusion

THC:CBD is increasingly recognised as a valuable option in the management of spasticity in ALS. The analysis of real world data, derived from a platform-based patient registry, demonstrated a wide range in the dose of THC:CBD for the treatment of ALS-related spasticity. Obviously, THC:CBD was used on the basis of individual patients’ needs and preferences. Furthermore, severe spasticity was associated with an increased number of daily THC:CBD actuations and a stronger recommendation rate on the NPS score as compared to patients with mild spasticity. Overall, patients reported outcomes as assessed by TSQM-9 revealed a high treatment satisfaction with THC:CBD. The results of our study suggest that THC:CBD may serve as an important addition to the spectrum of treatment options of spasticity in ALS. However, an important selection bias, and by that means limitation of the study, has to be considered as a 40% of patients (*n* = 16) discontinued THC:CBD treatment during the observation period. Further studies are warranted to confirm our results and to address the many still open issues in the therapeutic potential of THC:CBD in this disorder.

## Additional file

Additional file 1: Demographical and clinical characteristics data of the study participants. (PDF 54 kb)

## Data Availability

The datasets used and analysed during the current study are available from the corresponding author on reasonable request.

## Abbreviations

ALS: Amyotrophic lateral sclerosis
APST: Ambulanzpartner Soziotechnologie
CBD: Cannabidiol
MS: Multiple sclerosis
NPS: Net promotor score
NRS: Numeric rating scale
THC: Delta-9-tetrahydrocannabinol
TSMQ-9: Treatment satisfaction questionnaire for medication
