# Supplementary material for: Real world experience of patients with amyotrophic lateral sclerosis (ALS) in the treatment of spasticity using tetrahydrocannabinol:cannabidiol (THC:CBD)
Source: BMC Neurol. 2019 Sep 7;19:222. doi: 10.1186/s12883-019-1443-y (PMC6732193; doi:10.1186/s12883-019-1443-y)
Supplement: Supplementary file 1 — Demographical and clinical characteristics data of the study participants. (PDF 54 kb) [file 12883_2019_1443_MOESM1_ESM.pdf]

Additional file 1: Demographical and clinical characteristics data of the study participants

Patient ID	Age (years)	Gender	Type of onset	Disease duration	ALS-FRS	FTLD	Discon-tinuation	Antispas-modic me-dication
1	87	F	S	89	11	no	yes	BAC
2	83	F	S	33	20	no	yes	BAC
3	68	F	S	29	0	yes	no	no
4	49	F	S	50	30	no	yes	no
5	49	F	S	61	23	no	no	no
6	74	F	S	56	29	no	no	no
7	67	F	S	76	21	no	no	no
8	42	F	S	72	17	no	yes	no
9	72	F	S	43	23	no	no	BAC
10	34	M	S	65	14	no	no	BAC
11	70	M	S	123	15	no	yes	no
12	49	F	S	40	15	no	no	no
13	71	M	S	42	14	yes	yes	BAC
14	69	M	S	10	25	no	no	BAC, TIZ
15	77	F	S	99	13	no	yes	no
16	61	F	S	27	16	no	unknown	unknown
17	66	F	B	74	29	no	no	BAC
18	76	F	S	25	23	no	unknown	unknown
19	37	F	S	137	25	no	yes	no
20	66	F	S	16	32	no	no	no
21	47	M	S	10	23	yes	no	BAC
22	36	M	S	49	22	no	no	BAC
23	51	F	S	158	14	no	yes	no
24	55	F	S	38	27	no	no	no
25	40	F	S	18	28	no	no	no
26	33	F	S	14	36	no	no	no
27	49	M	S	40	14	no	no	no
28	44	F	S	244	38	no	no	no
29	77	M	S	86	33	no	no	no
30	74	M	S	34	24	no	unknown	unknown
31	45	F	S	39	8	no	yes	no
32	56	M	S	7	39	no	no	no
33	57	F	S	41	42	no	no	no
34	35	F	S	27	unknown	no	no	no
35	27	M	S	unknown	unknown	no	unknown	unknown

36	77	M	S	55	29	no	no	TIZ
37	38	M	S	41	12	no	yes	BAC
38	59	M	S	82	18	no	no	no
39	52	F	B	29	unknown	no	no	no
40	52	M	S	39	34	no	yes	no
41	72	M	S	67	28	no	yes	no
42	58	M	S	111	28	no	yes	no
43	60	M	S	36	33	no	yes	no
44	60	M	S	78	16	no	yes	no

F = Female; M = Male; S = Spinal; B = Bulbar; BAC = Baclofen; TIZ = Tizanidin; FTLD = Frontotemporal lobe degeneration
